# Residual Impact Performance of ECC Subjected to Sub-High Temperatures

**DOI:** 10.3390/ma15020454

**Published:** 2022-01-07

**Authors:** Raad A. Al-Ameri, Sallal Rashid Abid, Gunasekaran Murali, Sajjad H. Ali, Mustafa Özakça, Nikolay Ivanovich Vatin

**Affiliations:** 1 Department of Civil Engineering, Faculty of Engineering, Gaziantep University, Gaziantep 27310, Turkey; raada.alameri@gmail.com (R.A.A.-A.); ozakca@gantep.edu.tr (M.Ö.); 2 Department of Civil Engineering, College of Engineering, Wasit University, Kut 52003, Iraq; sajhali.wasit@gmail.com; 3 School of Civil Engineering, SASTRA Deemed University, Thanjavur 613401, India; murali_22984@yahoo.com; 4 Peter the Great St. Petersburg Polytechnic University, 195251 St. Petersburg, Russia; vatin@mail.ru

**Keywords:** ECC, repeated impact, high temperatures, ACI 544-2R, drop-weight, impact ductility

## Abstract

Despite the fact that the mechanical properties of Engineered Cementitious Composites (ECC) after high-temperature exposure are well investigated in the literature, the repeated impact response of ECC is not yet explored. Aiming to evaluate the residual impact response of ECC subjected to sub-high temperatures under repeated drop weight blows, the ACI 544-2R repeated impact test was utilized in this study. Disk impact specimens (150 mm diameter and 64 mm thickness) were prepared from the M45 ECC mixture but using polypropylene fibers, while similar 100 mm cube specimens and 100 × 100 × 400 mm prism specimens were used to evaluate the compressive and flexural strengths. The specimens were all cast, cured, heated, cooled, and tested under the same conditions and at the same age. The specimens were subjected to three temperatures of 100, 200 and 300 °C, while a group of specimens was tested without heating as a reference group. The test results showed that heating to 100 and 200 °C did not affect the impact resistance noticeably, where the retained cracking and failure impact numbers and ductility were higher or slightly lower than those of unheated specimens. On the other hand, exposure to 300 °C led to a serious deterioration in the impact resistance and ductility. The retained failure impact numbers after exposure to 100, 200, and 300 °C were 313, 257, and 45, respectively, while that of the reference specimens was 259. The results also revealed that the impact resistance at this range of temperature showed a degree of dependency on the compressive strength behavior with temperature.

## 1. Introduction

The conventional normal strength concrete has been a perfect building solution since the spread of Portland cement production. However, with the developments in building materials technology, new concrete types that possess better characteristics were introduced. The need for cementitious materials that can absorb high plastic energy before failure was the reason to design and introduce what is known as Engineered Cementitious Composites (ECC). ECC is a kind of flowable concrete that has perfect fresh properties and superior plastic deformation resistance. This material was first introduced by Victor Li [1,2], who worked on the design of ECC that can sustain high deformations by virtue of multi-cracking behavior [3,4]. The material includes a much higher content of cementitious materials than conventional concrete that has a similar compressive strength, where in addition to cement, other cementitious materials such as fly ash and silica fume are used with significant contents. For instance, the basic M45 ECC mixture includes 570 kg/m^3^ of cement, while the content of fly ash is 120% of cement content [5]. The presence of such contents can help reach the required fresh workability and the crack resisting characteristics. The use of very fine silica sand instead of the fine and coarse aggregates is also another reason for the material′s behavior, while the existence of synthetic fibers can assure the active resistance to crack opening and propagation, which shifts the behavior to the multi-cracking mode. Numerous studies were conducted during the last two decades to study the properties of ECC under the different kinds of loads, to explore the effect of different types of materials and fibers on the mixture characteristics, and to evaluate the material behavior under extreme conditions [6,7,8,9,10,11,12].

Impacts from falling masses on beams and slabs or due to the collision of moving cars on columns or walls are types of accidental non-design loads. Such loads can cause serious localized damage that may influence the member’s integrity or affect a part of the structure. The effect becomes more serious if these impacts are repeated several times. Examples of repeated impacts are the columns of parking garages that are subjected to daily high traffic with the possibility of repeated impacts by moving vehicles [13]. The tire impact of the landing aircraft on the airport runways is also another example of repeated impacts [14]. The water velocity reducing elements in hydraulic structures such as chute blocks in stilling basins are continuously subjected to repeated impacts from the high-velocity water and the carried debris [15]. Therefore, the performance of concrete materials and structural members under the influence of impact loads was the focus of research for decades. Several testing procedures are available to evaluate the different impacting loads depending on the velocity of the impactor and its direction. For instance, high strain rate tests, such as the projectile testing method, evaluate the performance of a structural member targeted by any kind of projectiles in war cases, conflict areas, or terrorist attacks [16,17]. On the other hand, tests such as drop-weight impact and Charpy pendulum test are more frequently used to assess the impact resistance of materials. However, these tests do not consider the effect of a repeat of impacts, while the ACI 544-2R [18] introduces a very simplified procedure to evaluate the material response to repeated falling impacts qualitatively. The test does not require measurements of load, deformation, or vibration, where only the count of impact blows is considered in the procedure. This test was recently facilitated by several researchers for the purpose of comparing the influence of adopting different materials and fiber types on the impact performance of concrete. The impact performance of concrete with polypropylene fibers [19,20,21,22], micro-steel fibers [23], steel fibers [24,25,26], carbon fibers [27,28], natural fibers [29], and hybrid fibers [30,31,32,33] was investigated recently. Significant research works were recently conducted to study the influence of fiber type, fiber dosage, fiber distribution, and intermediate fiber meshes on improving the impact resistance of preplaced aggregate concrete with single, double, and triple layers [34,35,36,37,38,39]. On the other hand, the research on the repeated impact behavior of ECC is very limited in the literature. The influence of fly ash, silica fume, slag, and metakaolin in different combined dosages on the repeated impact performance of ECC was investigated by Ismail et al. [40], where increasing the dosages of fly ash and metakaolin was found to give the highest impact numbers.

Despite the technological advances in fire control systems, trusted fire records reported thousands of accidental structural fires every single year all over the world [41]. Owing to the non-stop advances in construction materials technology, research on this topic is always required to help engineers make their decisions about the probable post-fire occupation of fire-exposed structures [42,43]. Extensive research works are available on the effect of high temperatures on the residual mechanical properties of concrete after fire exposure and post-fire structural evaluation of structures [44,45]. The changes of the concrete microstructure after reaching the different levels of temperature control the percentage residual strength. The pore water vaporization beyond 100 °C was reported to mostly have a positive effect on the compressive strength [46,47,48,49], while the serious strength reduction starts beyond 300 to 400 °C, where the chemical decomposition of calcium silicate hydroxide strongly deteriorates the material’s microstructure [50,51,52,53,54]. The influence of fire exposure on the compressive strength, tensile strength, flexural strength, shear strength, and elastic modulus of different concrete types was extensively studied in the literature [55,56,57,58,59]. Similarly, some previous studies were conducted to investigate the post-fire impact performance of concrete. However, most of them did not consider the repeated impact scenario, while very few researches were conducted using the repeated impact procedure. Mehdipour et al. [60] carried out mechanical and repeated impact tests on concrete specimens subjected to temperatures up to 600 °C. They investigated the effect of the partial replacements of cement by metakaolin and coarse aggregate by crumb rubber. Their results revealed a destructive effect of high temperatures on the retained impact numbers, where specimens exposed to 600 °C cracked after one impact. Al-Ameri et al. [61] studied the ACI 544-2R repeated impact performance of normal weight-normal strength concrete after exposure to high temperatures. The results showed that the effect of 100 °C on the retained impact numbers was minimal, while the residual impact strength after heating to 600 °C was less than 4%. On the other hand, some researches on the residual mechanical properties of ECC after exposure to fire temperatures was also conducted by previous researchers [62,63,64,65,66,67,68].

From the above-introduced literature survey, it is obvious that very limited literature is currently available on the repeated drop-weight impact performance of ECC. Similarly, studies on the residual repeated impact strength of all concrete types after high temperatures are rare. However, to the best of the authors’ knowledge, no previous research was conducted to evaluate the residual repeated impact strength of ECC exposed to elevated temperatures. Therefore, the experimental study presented in this research was directed to highlight this uncovered issue and fill this gap of knowledge. For this purpose, ECC disk specimens were exposed to sub-high temperatures, and their residual repeated impact strength was investigated using the procedure of ACI 544-2R [18].

## 2. Experiential Study

### 2.1. Materials and Mixtures

The M45 ECC basic mixture [5] was adopted with the same original material quantities but using polypropylene (PP) fibers instead of polyvinyl alcohol (PVA) fibers. In the cubic meter of this mixture, 570 kg of Portland cement was used in combination with 684 kg of fly ash, which means that the cement/fly ash ratio was 1.2. The 2 materials composed the high content binder of this mixture, while the only filler used was fine graded silica sand, respectively. The filler/cement ratio was 0.8, where 455 kg of silica sand was utilized in the mixture with 330 kg of mixing water and 4.9 kg of super-plasticizer. The PP fibers were used at a volumetric content of 2%.

Type I (class 42.5R) ordinary Portland cement was adopted with the chemical characteristics and physical properties shown in Table 1, which also lists the main characteristics of the fly ash. On the other hand, graded silica sand (0.08 to 0.25 mm) from Sika*^®^* with a 1500 kg/m^3^ bulk density was used as the filler of the mixture. To assure the required fresh workability of ECC, ViscoCrete 5930-L produced by Sika*^®^* was mixed with the mixture’s water. The length and diameter of the used PP fiber were 12 and 0.032 mm, while its density was 910 kg/m^3^. On the other hand, the tensile strength and elastic modulus of the used PP fiber were 400 and 4000 MPa, respectively.

### 2.2. Tests and Procedures

Cube specimens with a side length of 100 mm were used to conduct the compressive strength test, while prism specimens with 100 mm square cross-section, 300 mm span, and 400 mm length was used to conduct the four-point flexural test to obtain the modulus of rupture (MOR). On the other hand, disk specimens were used per the recommendations of ACI 544-2R [18] to carry out the repeated impact tests. The specimens were 150 mm in diameter and 64 mm in depth. For each temperature level, 6 cubes, 6 prisms, and 6 disks were cast and cured in water containers for 28 days.

**Figure 1:**
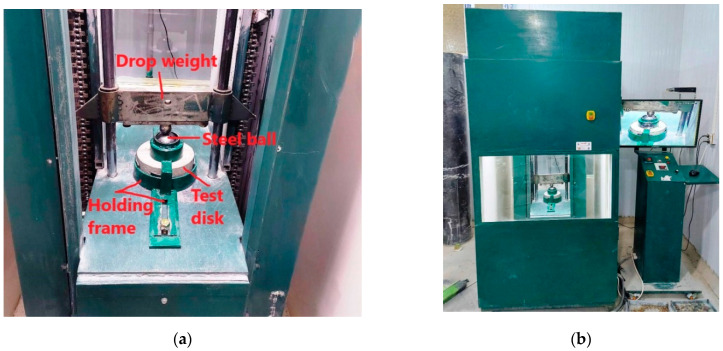
The impact testing (**a**) disk specimen’s holding apparatus (**b**) testing machine.

The impact specimens were tested under a falling weight of 4.54 kg and a falling depth of 457 mm as recommended by ACI 544-2R. The recommendations require that a steel ball of approximately 64 mm is placed on the top of the disk specimen and transfer the impacts from the falling weight to the specimen. The steel ball and the specimens should be held in their positions during the test using a special steel frame and lugs to prevent the rebound of the falling weight. As shown in Figure 1a, all these requirements were followed as recommended, where the impact tests were conducted using the specially made automatic falling mass testing machine shown in Figure 1b. The ACI 544-2R procedure recommends continuing to impact the specimens using the free-falling weight until the cracking of the top surface, where the number of impacts till this point is recorded as the cracking impact number. Then, the impact is resumed until the failure of the specimens by crack opening and fracture, at which the number of impacts is recorded as the failure impact number. To better observe the cracking and failure of the impact specimens, it is shown in Figure 1b that a high accuracy camera was used to observe the surface condition of the tested specimen during the test. This test is known for its high variability, which implies the use of a larger number of specimens to control the variation of the results. Therefore, 6 impact disk specimens were used at each temperature level. It should be noticed that this test was defined as a qualitative test and was not intended to give exact numbers about the impact strength of concrete.

### 2.3. Furnace Heating

**Figure 2:**
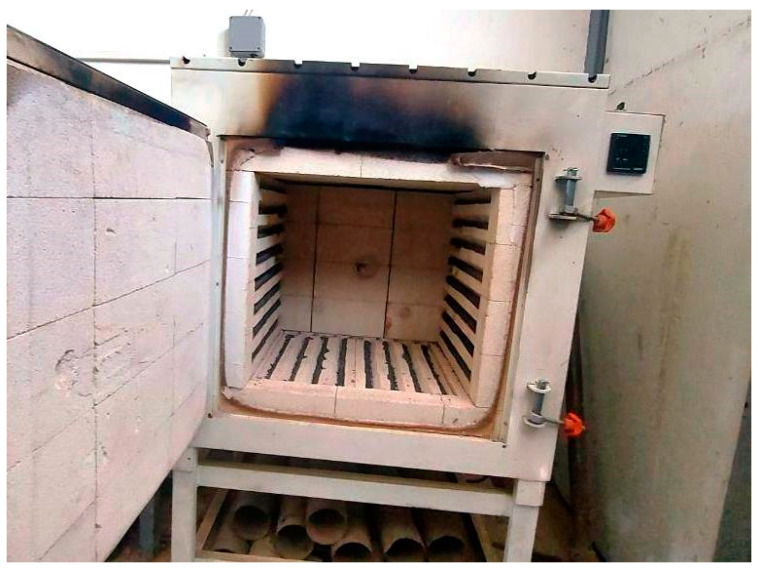
The interiors of the electric furnace.

**Figure 3:**
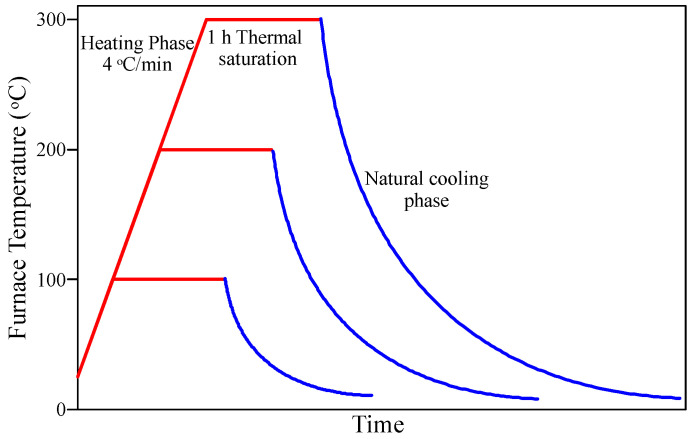
Furnace temperature-time relationships of the desired temperature levels.

A day before testing, the specimens were dried for 24 h using an electric oven at a temperature of 105 °C to prevent the unfavorable explosive failure inside the furnace. The heating of the specimens to the desired levels of temperature was conducted using the electrical furnace shown in Figure 2. In addition to the room temperature, the specimens were subjected to three levels of sub-high temperatures, which were 100, 200, and 300 °C. The heating rate of the furnace was kept approximately constant at 4 °C/min. As the desired temperature was reached, the furnace temperature was fixed for 60 min to thermally saturate the specimens at this temperature. Then, the furnace door was opened, and the specimens were naturally air-cooled until testing time. The regimes of heating and cooling of the 3 temperature levels are illustrated in Figure 3.

## 3. Compressive Strength Results

**Figure 4:**
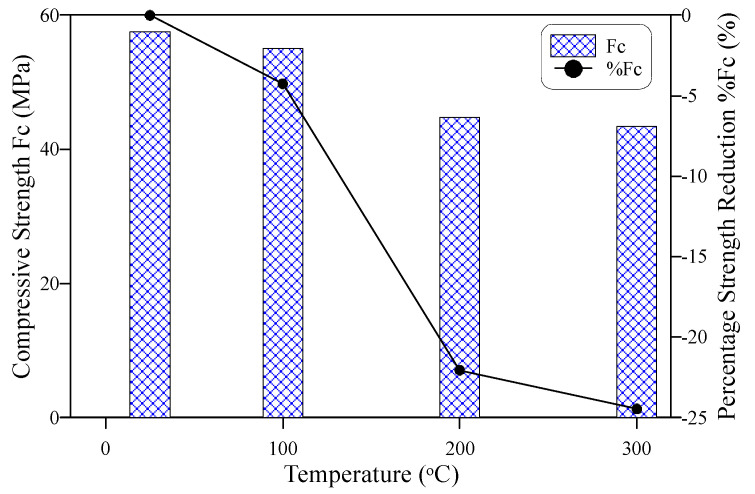
Compressive strength-temperature behavior.

As preceded, compressive strength cubes were cast, cured, heated, and tested at the same conditions as their corresponding impact test specimens. Figure 4 illustrates the influence of heating to the adopted three temperature levels on the compressive strength of the ECC specimens. Previous studies showed that the concrete compressive strength of ECC [69,70,71] increased in some cases after exposure to temperatures in the range of 100 to 300 °C, or exhibited a partial recovery after exposure to 300 °C. Such behavior did not occur in this study, where the compressive strength of ECC exhibited a continuous decrease as temperature increased. However, the strength reduction after exposure to 100 °C was limited to approximately 4%, as shown in Figure 4, where the compressive strength decreased from 57.5 to 55.0 MPa. Another notice is that the strength reduction at 200 °C was comparable to that at 300 °C, where the strength reduced to 44.8 and 44.4 MPa, respectively, exhibiting respective percentage decreases of 22 and 24.4%. A similar continuous reduction of compressive strength of ECC with temperature was also reported by previous studies [62,63].

## 4. Flexural Strength Results

**Figure 5:**
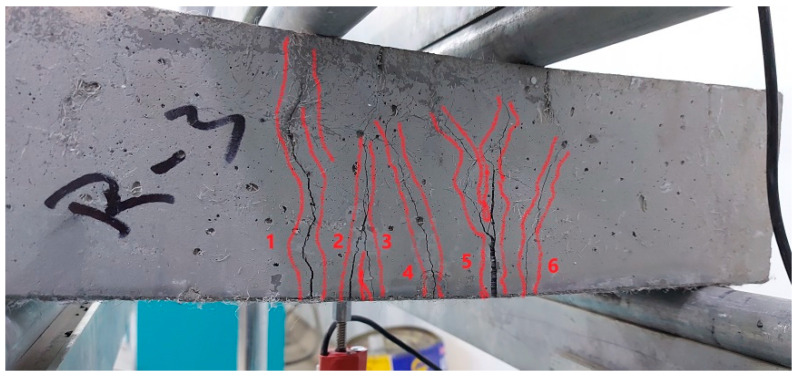
Multi-cracking failure of tested prisms.

**Figure 6:**
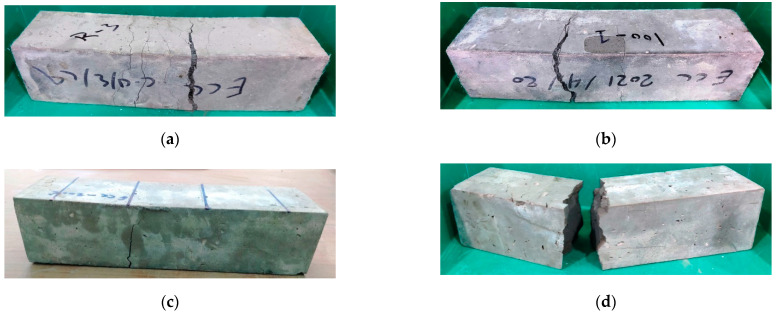
Failure of tested prisms exposed to different temperatures (**a**) Reference R, (**b**) 100 °C, (**c**) 200 °C, (**d**) 300 °C.

The flexural strength was tested using the four-point bending setup to determine the modulus of rupture (MOR). The unheated ECC specimens exhibited the multi-cracking ductile behavior as shown in Figure 5, where all specimens showed more than five cracks that extended from the bottom surface of the beam specimen vertically towards the top-loading points as the load was increased. Finally, one of these cracks was opened wider, leading to the failure of the specimen. This cracking behavior assures the ductile performance of the adopted PP-based ECC. A similar cracking and failure performance was also recorded for the specimens exposed to 100 °C as depicted in Figure 6b. However, the number of cracks was lesser than those in the unheated specimens. After exposure to 200 °C, the specimens exhibited a brittle failure with only one crack that widened quickly under the loading increase (Figure 6c), while the specimens exposed to 300 °C exhibited a clear sudden brittle failure similar to that of normal concrete as shown in Figure 6d. Another notice is that the visual examination after failure revealed that the PP fibers were clearly bridging the two sides of the cracks of the unheated specimens and those exposed to 100 °C, which explains their ductile behavior. On the other hand, such bridging activity was not observed for specimens exposed to higher temperatures due to the melting of the PP fibers at slightly below 200 °C, which in turn explains the brittle failure of these specimens.

**Figure 7:**
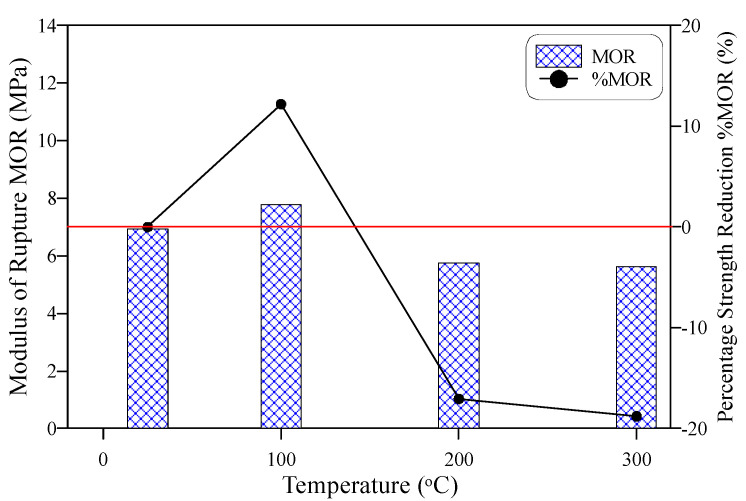
Modulus of rupture-temperature behavior.

**Figure 8:**
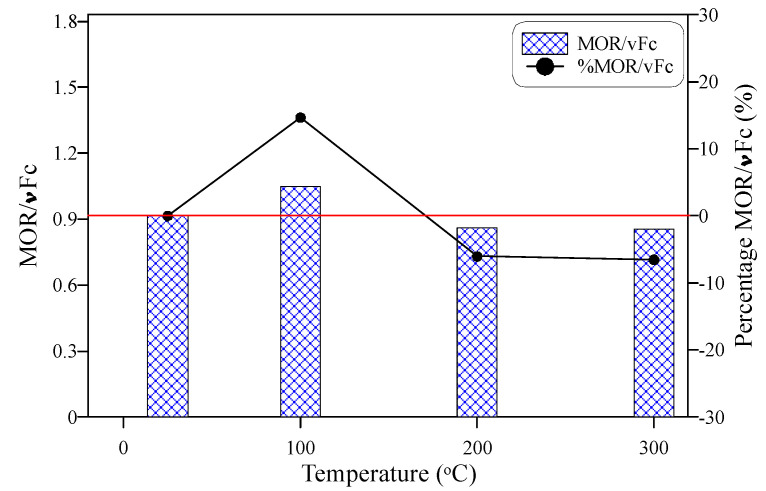
Behavior of normalized modulus of rupture with temperature increase.

Figure 7 shows the behavior of MOR with temperature increase. Similarly, the figure shows the percentage difference in MOR compared to the reference unheated MOR record. It can be seen in the figure that the MOR of the tested PP-ECC was not significantly affected by heating to temperatures up to 300 °C. The recorded MOR of the reference unheated specimens was 6.94 MPa, while after exposure to 100 °C, this value increased to 7.78 MPa, recording a percentage increase of 12.2% compared to the reference record. However, after exposure to 200 and 300 °C, MOR reduced by 17 and 18.8%, respectively, as clearly depicted in Figure 7. Thus, the MOR was kept within an approximately ± 20% difference with the unheated specimens. The increase in strength at 100 °C can be attributed to the densification of the material due to the vaporization of free pore water, while the melting of fibers after 200 °C left open micro-tubes inside the matrix that reduced its stiffness and weakened its structure [63,72]. Consequently, the strength was less than that before heating. The obtained trend of results in this study agrees with records reported by previous researchers [64]. However, the percentages of increase after 100 °C exposure and decrease after exposure to 200 °C and higher were different owing to the different mixtures, fiber types, heating regimes, and other reasons. For instance, Yu et al. [68] reported a percentage decrease of approximately 50% for ECC reinforced with PVA fibers after exposure to 200 °C, while the percentage decrease at the same temperature was only 25% for specimens reinforced with both PVA and steel fibers. On the other hand, Wang et al. [71] reported a partial recovery at 100 °C after an initial decrease at 50 °C, followed by a significant decrease after exposure to temperatures of 200 °C and higher. To better visualize the effect of temperature on MOR taking into consideration its relation with the compressive strength, MOR was normalized by the square root of the corresponding compressive strength record at each temperature as depicted in Figure 8. As shown in the figure, the trend of the normalized MOR with temperature is quite similar to that of MOR. However, the percentage decrease values were affected by those of the compressive strength and reduced to approximately 6% after exposure to 200 and 300 °C. This means that compressive strength could partially affect the behavior of flexural strength after exposure to temperatures up to 300 °C.

## 5. Repeated Impact Strength Results

### 5.1. Impact Number Results

**Figure 9:**
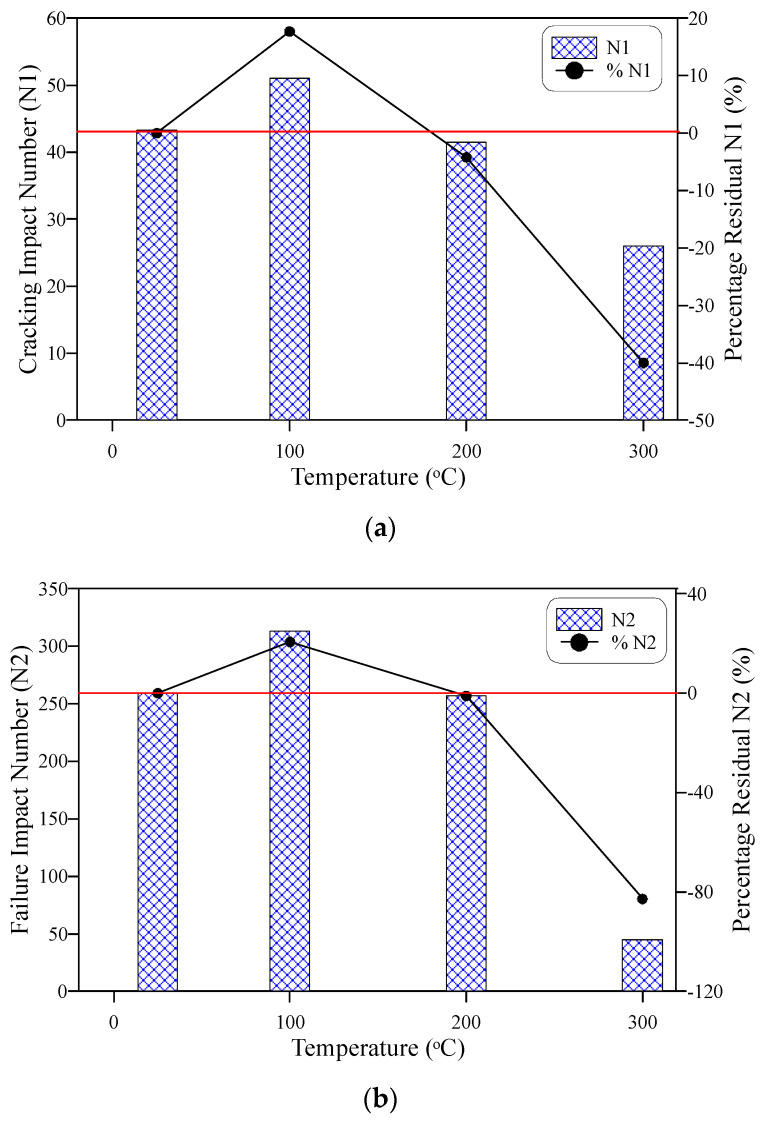
Impact number-temperature behavior (**a**) cracking number N1, (**b**) failure number N2.

The impact strength in this study is represented by the retained numbers of impact blows at cracking and failure stages using the ACI 544-2R repeated impact test [18]. Figure 9a shows the recorded cracking number (N1) at each temperature and the percentage residual of N1 based on the record of the unheated specimens. It is obvious in the figure that ECC specimens retained higher N1 after exposure to 100 °C than that of the reference unheated specimens. On the other hand, a slight decrease in N1 was recorded after exposure to 200 °C, while a serious decrease occurred after exposure to 300 °C. The retained N1 records after exposure to 100, 200, and 300 °C were 51, 41.5, and 26 blows, respectively, while the unheated reference N1 was 43.3. Hence, the cracking impact number increased by 17.7% after exposure to 100 °C and decreased by 4.2 and 40% after being heated to 200 and 300 °C, respectively.

Following a similar behavior with temperature increase, the failure impact number (N2) of specimens exposed to 100 °C was higher than the corresponding reference unheated N2, as shown in Figure 9b. This increase was also followed by a slight decrease after exposure to 200 °C. However, the decrease of N2 after heating to 300 °C was much higher than its corresponding N1, which reflects the much higher effect of heating at this temperature on the failure impact resistance than the cracking impact resistance. The retained N2 values were 313, 256.7, and 44.7 after exposure to 100, 200, and 300 °C, respectively, while the reference N2 was 259.3 blows. This means that N2 increased by approximately 21% after 100 °C exposure, while almost kept the same as the reference N2 with a slight decrease of 1% after exposure to 200 °C. On the other hand, a serious N2 drop of approximately 83% was recorded for the specimens heated to 300 °C.

The higher reduction after exposure to 300 °C is attributed to the microstructural changes that occurred at this temperature, where the complete melting of PP fibers left behind open micro-tubes that composed a network of connected continuous pores, which increased the porosity of the material and made it more brittle. Sahmaran et al. [62] indicated that the increase in PVA-ECC porosity was minimal (0.2%) before fiber melting (200 °C), while the complete melting of fibers increased the porosity to 5% and more after exposure to higher temperatures. Similarly, the increase in pore diameter was minimal after exposure to 200 °C, while the exposure to 400 °C resulted in an increase in pore diameter by about 50%, which explains the minor decrease in N1 and N2 after exposure to 200 °C and the significant decrease after exposure to 300 °C. Bhat et al. [73] reported an increase in the tensile strength of PVA-ECC after exposure to 100 °C, which was attributed to the initial moisture loss due to the free pore water evaporation (form larger pores and capillary pores) and the hydration of the free fly ash that was not hydrated during the curing period. This was in turn attributed to the large amount of fly ash used in ECC. It should also be noticed that fibers were still working at 100 °C, while the weakening of the bridging activity of fibers after exposure to 200 °C (due to the partial melting) and the extra drying activity of temperature led to the slight reduction in N1 and N2 at this temperature level. It was also reported [73] that the moisture loss was less than 3% after 100 °C exposure, while it was more than 7% after exposure to 200 °C.

**Figure 10:**
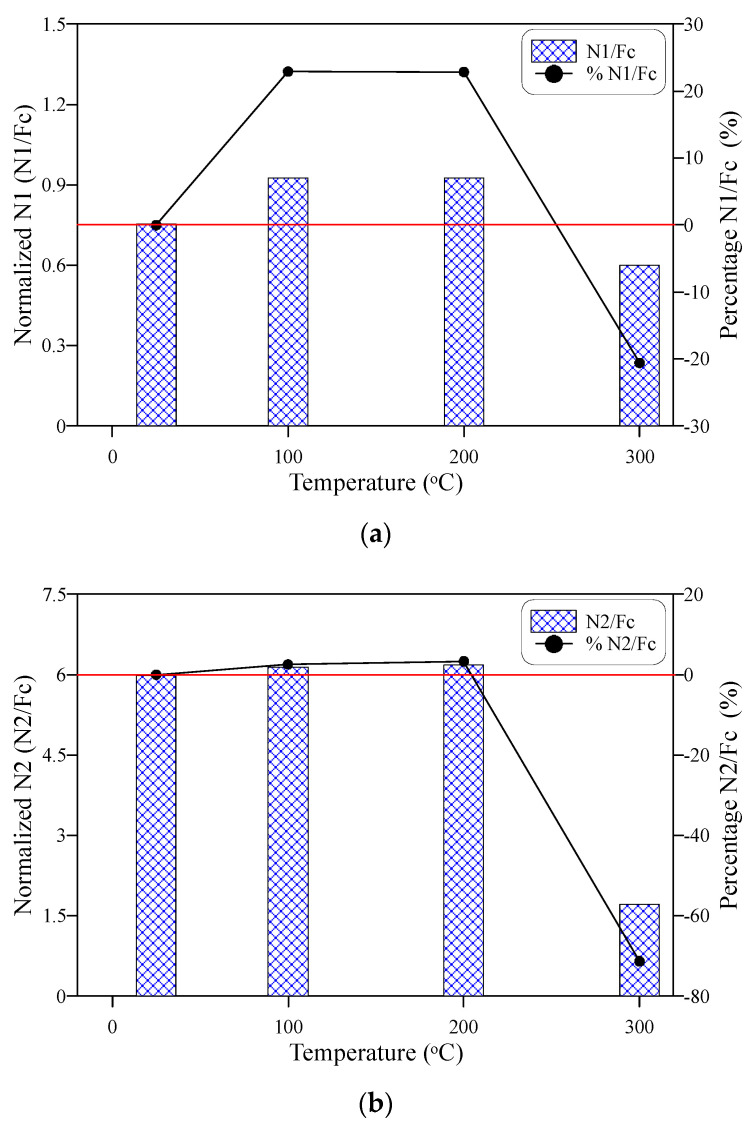
The behavior of normalized impact number with temperature increase (**a**) cracking number N1, (**b**) failure number N2.

To study the effect of compressive strength decrease with temperature on the response of impact numbers after temperature exposure, the impact numbers N1 and N2 were normalized by their corresponding compressive strength values at each temperature and presented in Figure 10a,b, respectively. It is obvious that the compressive strength was effective on the response on both N1 and N2, where the normalized responses exhibited differently from the corresponding responses of N1 and N2 (Figure 9a,b). Two differences can be distinguished between the responses of impact numbers and the normalized impact numbers. The first is that the normalized values of N1 at 100 and 200 °C were almost equal and thus as those of N2 and were both higher than the reference unheated normalized impact numbers, while the impact numbers (Figure 9) exhibited an increase at 100 °C and decreased at 200 °C. The second difference is that the percentage reductions after exposure to 300 °C of the normalized N1 and N2 (Figure 10) were less than those of N1 and N2 (Figure 9). The percentage residual normalized N1 values exhibited an approximate increase of 23 after exposure to 100 and 200 °C and an approximate decrease of 21% at 300 °C, while the corresponding percentages of N2 were approximately 3, 3, and −71% after exposure to 100, 200, and 300 °C, respectively. These differences between the behaviors of impact records and their normalized values with temperature reflect the dependency of impact strength on the compressive strength when exposed to temperatures up to 300 °C.

### 5.2. Failure of Impact Specimens

**Figure 11:**
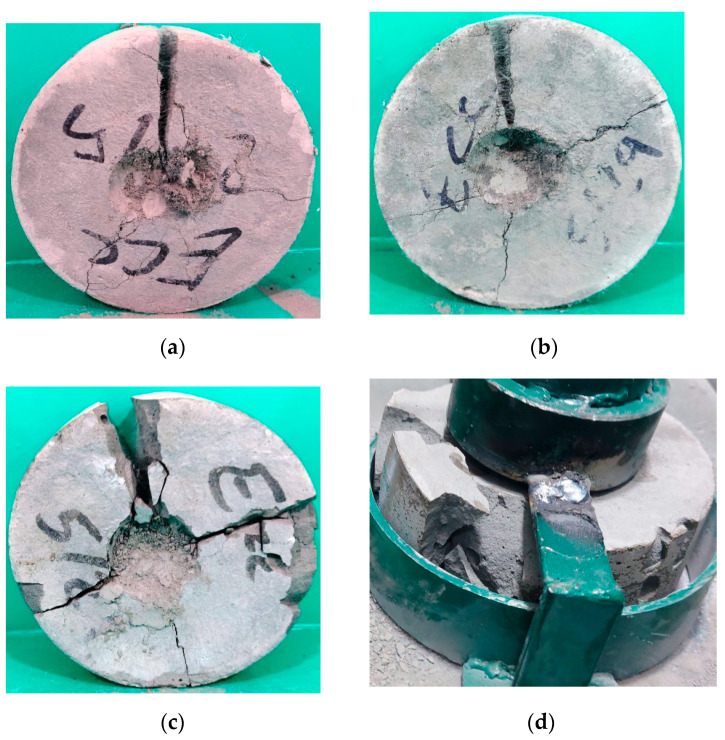
Tested disc specimens after failure (**a**) Reference R, (**b**) 100 °C, (**c**) 200 °C, (**d**) 300 °C.

The behavior of repeated impact numbers with temperature is also confirmed by the failure patterns of the tested specimens. Figure 11 shows pictures of tested ECC disk specimens that were exposed to the investigated temperature levels. It is obvious in Figure 11a that the unheated reference specimens exhibited the usual ductile failure of fiber-reinforced specimens, which is associated with a central fracture zone and multi-surface cracking. Owing to the test setup, the drop weight concentrically impacts the steel ball that is placed on the center of the top surface. With the increase of impact number, the top cementitious layer of this central zone (under the steel ball) starts cracking, which is followed by the fracturing of the below-surface layer. At this stage, the fibers try to work as a shield that composes what is termed as the shadow zone that protects the shaded cement matrix [74]. However, this shadow zone cannot stand long under the repeated impacts, especially since the used fiber was synthetic and not steel fibers. Therefore, the central surface impact zone completely fractures, as shown in Figure 11a, and the stress transfers to the outer perimeter of this zone leading to the surface cracking. At this stage, the bridging action of fibers becomes fully functional to slow down the crack propagation and widening. However, with the increasing of impact stresses, the fiber crack arresting capability is weakened and finally is broken, leading to further surface cracking and failure by the opening of one or more cracks [13].

The specimens exposed to 100 °C exhibited the same ductile cracking and failure behavior recorded for the reference specimens, where as shown in Figure 11b, the surface cracking and central fracturing are very similar to those that occurred on the surface of the unheated specimens (Figure 11a). Similarly, both figures show the presence of bridging PP fibers across the major opened cracks. This result supports the recorded N1 and N2 values that were not negatively affected by the exposure to 100 °C. On the other hand, the specimens exposed to 200 °C exhibited central fracturing and multi-cracking, as shown in Figure 11c. However, PP fibers were observed neither in the central fracture zone nor across the widened cracks, which is attributed to the partial melting of PP fibers at this temperature. This failure type can be classified to be a transition state between ductile and brittle failure cases. Oppositely, the specimens exposed to 300 °C exhibited a rapid brittle failure after cracking that was not associated with a central fracturing zone, as shown in Figure 11d. This behavior is in complete agreement with the rapid deterioration of N2 that decreased by more than 80% of its reference number, where the specimens failed after less than 20 blows after the initial cracking.

### 5.3. Impact Ductility

One of the structural physical quantities that are used to define the response of flexural members under loads is ductility, which is a kind of quantitative measurement to define the capacity of the member to sustain plastic deformations [75]. A similar definition was also used in previous studies [25,35,37] to estimate the plastic range of repeated impact that the specimen can withstand before failure. The impact ductility index (D) can thus be defined as the ratio of the failure impact number to the cracking impact number (D = N2/N1), which agrees with the original definition of flexural ductility, where it is defined as the ratio of the ultimate deflection to the deflection at the yielding of the tension steel bars [76].

**Figure 12:**
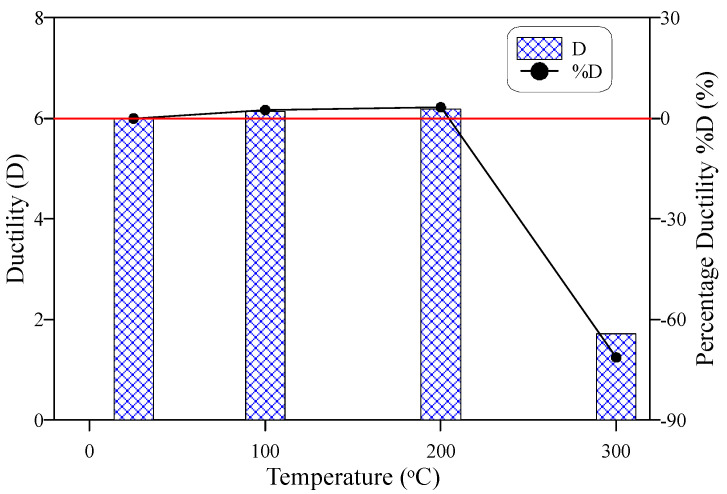
Impact ductility-temperature behavior.

As preceded in the previous sections, N1 and N2 records did not suffer serious deterioration after exposure to 100 and 200 °C, where these values were slightly higher at 100 °C and slightly lower at 200 °C than those of the reference specimens. This behavior was reflected on the impact ductility, where as shown in Figure 12, the ductility index values were almost the same at the three temperatures, which were 6.0, 6.1, and 6.2 at room temperature, 100 °C and 200 °C, respectively. These results reflect the ability of specimens heated to temperatures up to 200 °C to withstand significant impact numbers after surface cracking. Oppositely, the ductility index of the specimens exposed to 300 °C deteriorated significantly to only 1.7, owing to the serious microstructural effects of temperature and melting of fibers that led to a sharp drop in N2.

**Figure 13:**
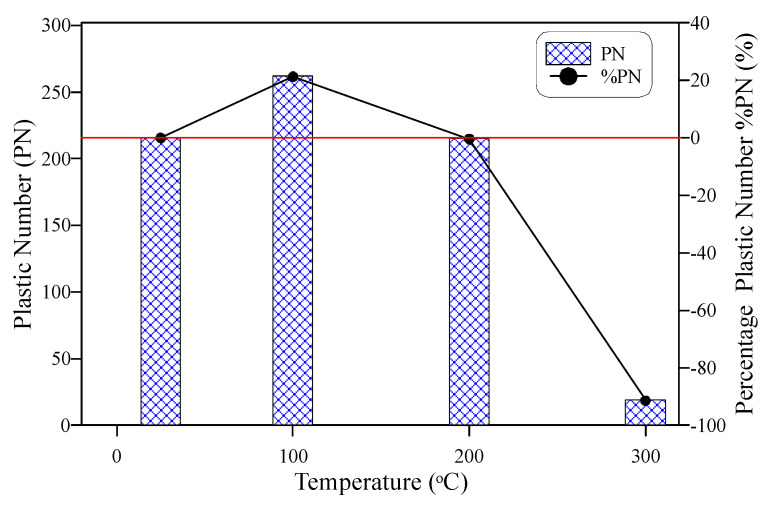
Plastic impact number-temperature behavior.

Figure 13 shows the ductility of the tested specimens using a different definition, which is the plastic impact number (PN) that simply represents the difference between N2 and N1. Hence, it is the number of impacts absorbed by the specimen after cracking. This number is sometimes required to highlight the weakness of impact ductility to compare between different groups of specimens. Where in some cases, both N1 and N2 drop sharply to very low numbers, that is, although there are very few differences between N1 and N2, the ductility index was higher than expected. Figure 13 shows that PN of the specimens heated to 200 °C equals that of the unheated specimens, while that of the specimens heated to 100 °C is noticeably higher than both. Noting that the ductility was slightly higher at 200 °C than at 100 °C (Figure 12), PN reflects the higher plastic potential of specimens heated to 100 °C than those heated to 200 °C. Another notice is that heating to 300 °C reduced the ductility index by approximately 71%, while the percentage reduction in PN was approximately 91%, which is noticeably higher. Thus, PN can be used as a secondary evaluation tool for ductility in the case of evaluating the residual repeated impact ductility.

## 6. Conclusions

Engineered cementitious composite disk specimens were cast in this research to evaluate the repeated impact response using the ACI 544-2R procedure, while cube and prism specimens were used to evaluate the compressive strength and modulus of rupture. The specimens were heated to three temperatures of 100, 200, and 300 °C, while a reference group of specimens was tested without heating. From the results obtained in this study, the followings are the concluded points,

1. Cracking (N1) and failure (N2) impact numbers increased after exposure to 100 °C compared to the unheated reference specimens, where N1 and N2 increased by approximately 18 and 21%, respectively. The impact resistance in terms of N1 and N2 of ECC was almost unaffected by the exposure to 200 °C, where the percentage decrease values of N1 and N2 were limited by approximately 4 and 1%, respectively. However, exposure to 300 °C led to a significant impact resistance deterioration, which was more severe at failure than at the cracking stage with percentage decrease values of 40 and 83%, respectively.
2. To evaluate the influence of compressive strength on the response of cracking numbers with temperature, N1 and N2 were normalized at each temperature level by the corresponding compressive strength records at these temperatures. The normalized N1 and N2 values reflected a noticeable dependency degree of the temperature-response of N1 and N2 on that of compressive strength. The normalized N1 and N2 were almost constant at 100 and 200 °C, and both were higher than the unheated reference values, which is different behavior than that of N1 and N2 with temperature. The reductions at 300 °C were also lower for the normalized N1 and N1 cases than their corresponding N1 and N2 records. The different behaviors of normalized impact numbers from those of impact numbers reflect the noticeable degree of dependency of impact resistance on the compressive strength after exposure to temperatures of 100 to 300 °C.
3. The specimens heated to 100 °C exhibited a ductile failure similar to that of the unheated specimens, which was characterized by the fracturing of the central zone of the top surface followed by surface multi-cracking and finally failed by the widening of one or more major cracks. Oppositely, the specimens exposed to 300 °C exhibited a sudden brittle failure without central fracturing or pre-failure multi-cracking. This behavior is attributed to the deterioration of the material microstructure and the complete melting of fibers. On the other hand, a kind of transition failure state between ductile and brittle failures was noticed for the specimens heated to 200 °C, where a weaker central fracturing zone was observed associated with wider cracks.
4. The ductility index was comparable for the unheated specimens and those subjected to 100 and 200 °C, which was approximately 6 with only a minor difference of about 3%. This result is attributed to the behaviors of N1 and N2 with temperature, where the exposure to these temperature levels did not lead to any significant deterioration in the impact resistance. However, the sharp drop of the failure impact number after exposure to 300 °C reduced the impact ductility significantly to less than 30% of the unheated value.

## Figures and Tables

**Table 1:** Characteristics of cement and fly ash.

Oxide (%)	Cement (%)	Fly Ash (%)
SiO_2_	20.08	56.0
Fe_2_O_3_	3.6	24.81
Al_2_O_3_	4.62	5.3
CaO	61.61	4.8
MgO	2.12	1.48
SO_3_	2.71	0.36
Loss on ignition (%)	1.38	5.78
Specific surface (m^2^/kg)	368	-
Specific gravity	3.15	2.20
Fineness (% retain in 45 μm)	-	28.99

## Data Availability

Not applicable.
